# *xrdPlanner*: exploring area detector geometries for powder diffraction and total scattering experiments

**DOI:** 10.1107/S1600577523011086

**Published:** 2024-02-02

**Authors:** Lennard Krause, Frederik Holm Gjørup, Mads Ry Vogel Jørgensen

**Affiliations:** a Department of Chemistry, Aarhus University, Langelandsgade 140, 8000 Aarhus C, Denmark; b MAX IV Laboratory, Lund University, Fotongatan 2, 225 94 Lund, Sweden; c iNANO, Aarhus University, Langelandsgade 140, 8000 Aarhus C, Denmark; University of Malaga, Spain

**Keywords:** X-ray diffraction, X-ray scattering, synchrotron facilities, software tools, experiment planning

## Abstract

*xrdPlanner* is a software package designed to aid in the planning and preparation of powder X-ray diffraction and total scattering beam times at synchrotron facilities. Many modern beamlines provide a flexible experimental setup and may have several different detectors available. In combination with a range of available X-ray energies, it often makes it difficult for the user to explore the available parameter space relevant for a given experiment prior to the scheduled beam time. *xrdPlanner* was developed to provide a fast and straightforward tool that allows users to visualize the accessible part of reciprocal space of their experiment at a given combination of photon energy and detector geometry. To plan and communicate the necessary geometry not only saves time but also helps the beamline staff to prepare and accommodate for an experiment. The program is tailored toward powder X-ray diffraction and total scattering experiments but may also be useful for other experiments that rely on an area detector and for which detector placement and achievable momentum-transfer range are important experimental parameters.

## Abstract 2

*xrdPlanner* is a software designed to assist in the planning of powder X-ray diffraction and total scattering experiments at synchrotron facilities. It provides a straightforward visualization of projected resolution intervals at different combinations of photon energy and detector geometry, and focus is put on an intuitive and fast presentation to facilitate live exploration of the available parameter space.

## 1. Introduction

Scattering experiments at synchrotron facilities have become more and more accessible and are already a routine for many researchers. However, a synchrotron beamline often offers much more experimental flexibility than X-ray instruments in a home laboratory (*e.g.* Vaughan *et al.*, 2020 ▸; Dippel *et al.*, 2015 ▸; Willmott *et al.*, 2013 ▸). This may leave, especially new, synchrotron users unable to take full advantage of the possibilities offered at a beamline. Moreover, the available information prior to a beam time is often limited to the X-ray energy and the type of detector. Often neglected are other parameters of the experimental setup, *e.g.* the degrees of freedom for detector movement and their ranges, and beamstop dimensions and distance.

More experienced users might have already established a *modus operandi*; however, even here the exploration of available experiment geometries might lead to potential optimization (Burns *et al.*, 2023 ▸). Here, we present a new free and open-source software tool, *xrdPlanner*, designed to help users plan their powder X-ray diffraction (PXRD) and total scattering (TS) experiments better.

**Figure 1:**
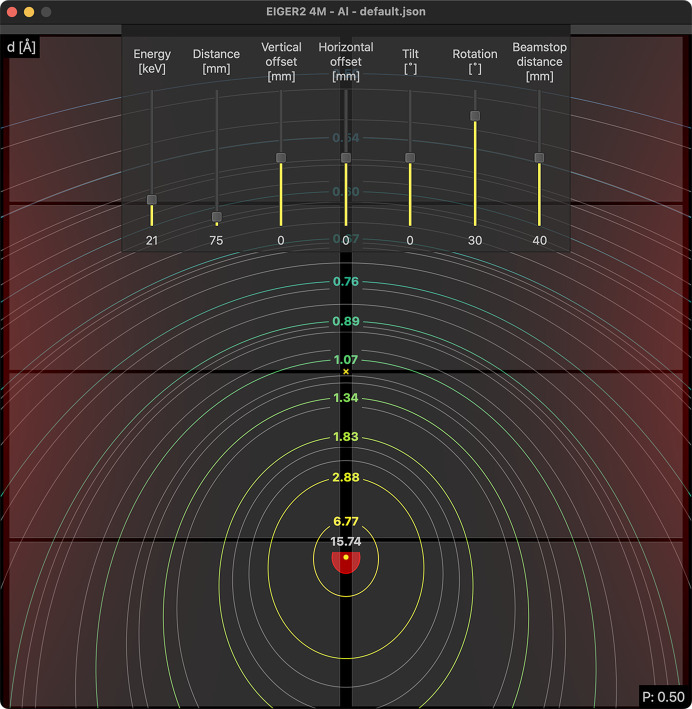
An example image showing an EIGER2 4M detector rotated 30° around a sample. The coloured contours display the resolution as *d* spacing in Ångstrom. The grey contours show the projected Debye–Scherrer rings of an aluminium standard sample from the *pyFAI* database (Kieffer *et al.*, 2020 ▸), the red area is the projection of the beamstop with the limiting resolution indicated by the grey label. The point of normal incidence (PONI) is indicated with a small cross (see also Fig. 4 ▸). The effect of polarisation is mapped onto the detector (red semi-transparent overlay) to indicate how large the effect will be.

The central idea behind the design of the program is to facilitate easy exploration of the possible experiment geometries, allowing the user to visually assess the achievable angular and azimuthal ranges for a given experimental setup. The core functionality is the projection and visualization of diffraction cones onto an area detector screen under various geometrical configurations and photon energies, all easily accessible through a user-friendly graphical user interface (GUI), see Fig. 1 ▸. The software is not intended for the visualization, analysis or processing of experimental data, where a plethora of excellent software is already existing. The target demographic is any users of PXRD and TS beamlines, independent of experience level, though any users or future users of area detectors might benefit, including students.

This article is meant to provide an overview and present the features of the software. For more details and a complete user guide, the interested reader is referred to the project GitHub page.

## 2. Description and features

The experimental setup in the program consists of the detector model, the sample-to-detector distance (SDD), the horizontal and vertical offset of the detector, the tilt of the detector, the rotation of the detector around the samples, and the photon energy. It can further include a model of the beamstop diameter and the sample-to-beamstop distance.

The detector type and model are selected in a drop-down menu. The most common detectors [including DECTRIS PILATUS3 (Broennimann *et al.*, 2006 ▸)/EIGER2 (Donath *et al.*, 2023 ▸)/PILATUS4, SACLA MPCCD (Kameshima *et al.*, 2014 ▸), Bruker Photon II, Varex Imaging and Rayonix MX-HS] are included and the detector database can be extended manually to add new detectors.

The photon energy and all the detector-geometry parameters are adjusted using a set of sliders in the fold-out menu at the top of the main window. From this input, the conic sections are calculated and visualized to give a live update.

A conic section is the curve generated by the intersection of the detector plane with the surface of a scattering-angle-dependent diffraction cone. Here, the beam and sample positions are kept fixed and point-like, but different detector geometries and X-ray energies affect the detectable part of the scattering. The contour of the conic section is well suited to visualize the recorded range of scattering angles upon change of detector type, photon energy and experimental geometry. Visualization and projection of the conic sections onto the detector area at different scattering angles or resolution intervals make experiment planning straightforward (coloured and labelled contours in Fig. 1 ▸). The contour lines can be labelled with either the scattering angle 2θ, the interplanar spacing *d*, the magnitude of the momentum-transfer vector *q* or the often-used sin(θ)/λ to express the resolution.

Besides the contours described above, it is also possible to project Debye–Scherrer cones for standard reference materials (SRMs, *e.g.* Si, Ni, LaB_6_) using a built-in library. The built-in SRMs use the *pyFAI* calibrant library (Kieffer *et al.*, 2020 ▸), which contains *d* spacings for the reflections of many common standard samples. The library is available from a drop-down menu and will project Debye–Scherrer cones (grey contours in Fig. 1 ▸) onto the detector plane alongside the resolution rings (coloured and labelled contours in Fig. 1 ▸).

**Figure 2:**
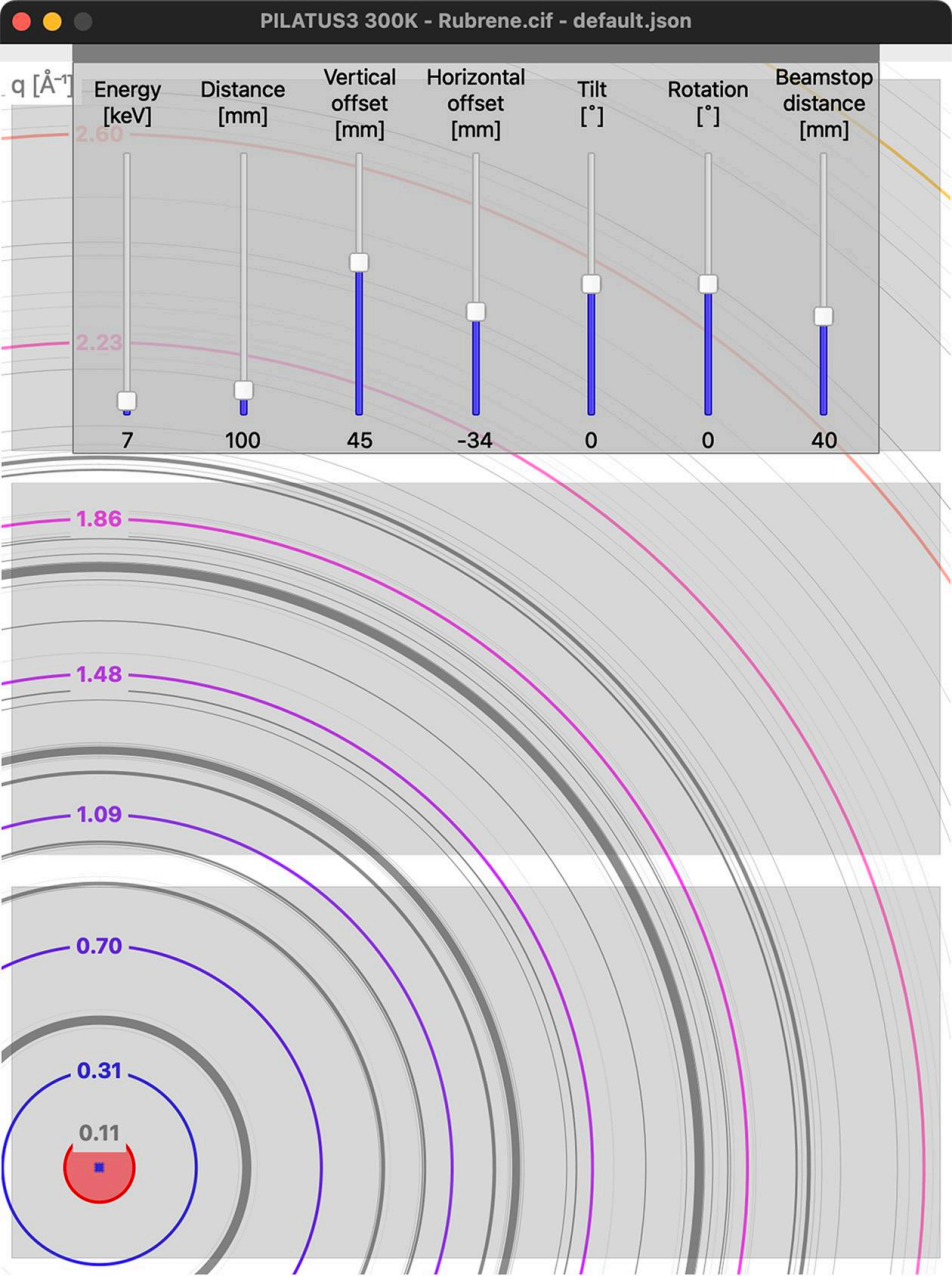
An example image showing a PILATUS3 300 K detector. The coloured contours display the resolution as the magnitude of the momentum-transfer vector *q* (Å^−1^). The grey contours show the projected Debye–Scherrer rings of a rubrene structure, calculated from the cif by *Dans_Diffraction* (Porter & Prestipino, 2023 ▸). The width is proportional to the intensity.

Visualization of other general samples is done by simple drag and drop of a Crystallographic Information File (cif; Hall *et al.*, 1991 ▸; Brown & McMahon, 2002 ▸) onto the main window. The program utilizes the *Dans_Diffraction* (Porter & Prestipino, 2023 ▸) Python package to calculate the intensity and *d* spacing of the reflections directly from the structure. The Debye–Scherrer rings are plotted, thus allowing the quick assessment of the required experimental geometry to guide the preparation of the experiments. The calculated intensity of each reflection is used to scale the width of the specific ring and makes the identification of a pattern more intuitive (see Fig. 2 ▸). The sample is assumed to be point-like, *i.e.* broadening of any kind is not included in the visualization. Clicking the conics will show the corresponding Laue indices.

Knowing how the expected scattering of the sample looks for a given geometry aids in deciding on an optimal experimental setup. This may be especially important for hybrid-pixel detectors that are made up of tiling of smaller modules with inactive gaps between them. Here, it is often necessary to consider where certain reflections will appear, to minimize or completely avoid overlap of a Debye–Scherrer ring and the detector gap. Similarly, it is possible to include visualization of polarisation and solid-angle effects. These are shown as semi-transparent overlays where the opacity is proportional to the size of the effects, see Fig. 1 ▸.

## 3. Use cases

**Figure 3:**
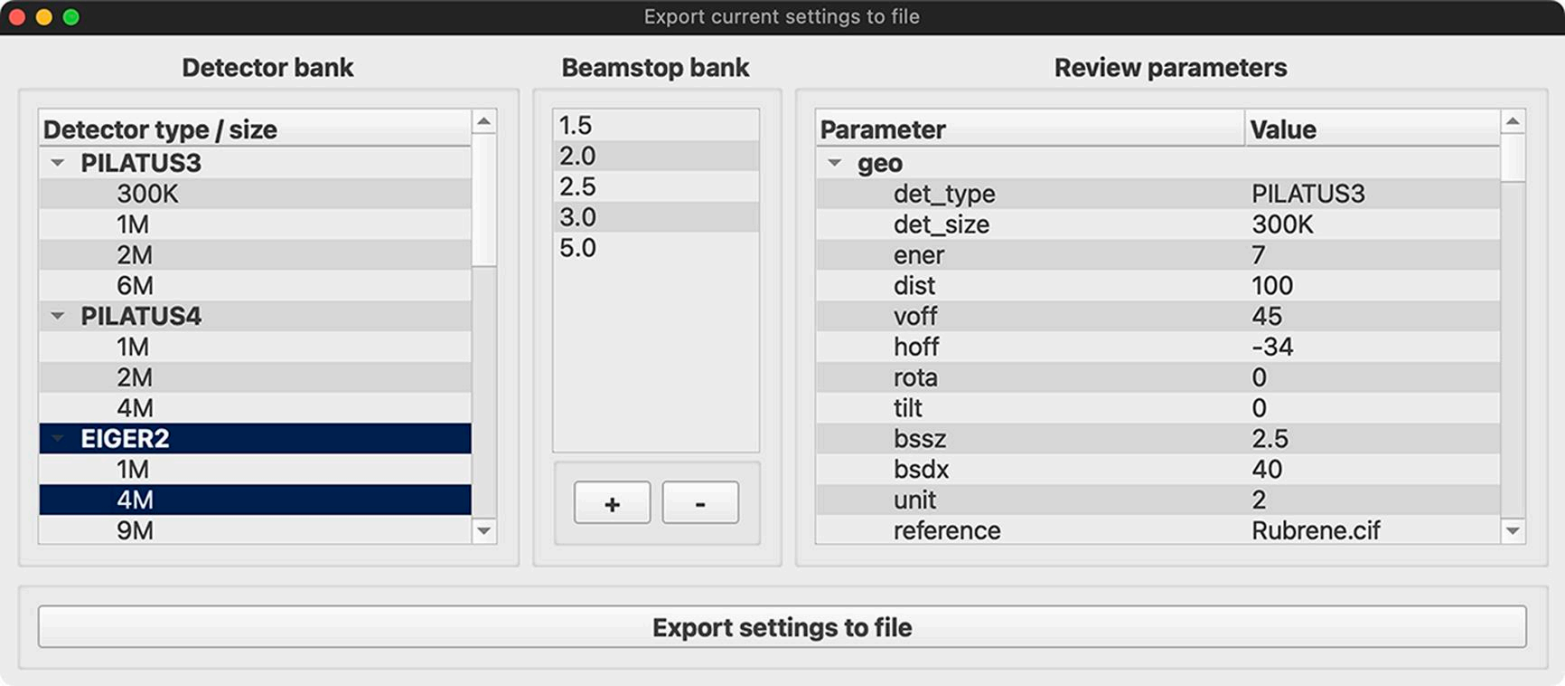
The export window allows the user to review and specify all parameters and limiting ranges of the settings file before export. Special emphasis was put on the detector bank, *e.g.* which detector models and types are available and the entries of the beamstop bank.

The software is intended to be used by both beamline staff and beamline users. The beamline staff can set up a model of their beamline, specifying the detector model, proper limits on all detector geometries, photon energy and beamstop dimensions to reflect the actual instrument. These settings can be stored in a JSON formatted text file that can be shared with the users either via the project GitHub page or, for example, the beamline’s website. By having the instrument model available, the beamline staff can easily plan and adjust experiments together with the users at the beamline. To facilitate the distribution of settings files from beamlines, the export window (see Fig. 3 ▸) serves as a guide to specify the characteristics of the available setup. It is possible to limit the available detectors and models by modifying the entries in the detector pool, add the available beamstop sizes, and set proper limits to the parameters. Tooltips add explanatory descriptions to the usage of each of the keywords.

The beamline user must make decisions regarding the sample environment and additional equipment when submitting the experiment proposal. Other key parameters to be assessed before the beam time are the required maximum scattering angle or, depending on the experiment, if specific diffraction rings are of particular interest. Allowing users to download the setup file for a particular beamline gives them the possibility to explore the experimental parameter space and find an optimal geometry, and avoids *ad* *hoc* decisions upon arrival. Moreover, if communicated to the beamline staff early, the chances of a successful beam time are expected to be higher.

## 4. Technical description

The software is based on *Python3* (Van Rossum & Drake, 2009 ▸) using the *PyQt6* framework. It is designed as a stand­alone application, but integration into existing *PyQt6* GUIs is straightforward. It relies on the *pyFAI* library (Kieffer *et al.*, 2020 ▸) for the SRM library and the *Dans_Diffraction* (Porter & Prestipino, 2023 ▸) Python library to read cifs and perform the *d* spacing and intensity calculations.

The GUI is readily customizable to blend in with existing software and offers the ability to edit configuration files, allowing users to adjust start-up defaults, layout, visuals and limits on parameters. A text-based JSON formatted settings file stores all the parameters, *e.g.* the energy range, beamstop size, degrees of freedom and ranges of motion of the detector. All parameter limits and step sizes can be adjusted or completely disabled, allowing the users to explore within the boundaries of a given experimental station.

A second JSON settings file contains the built-in detector systems. The most common detectors are already available for use but the database can be extended, and new custom-made detectors can be added as well.

**Figure 4:**
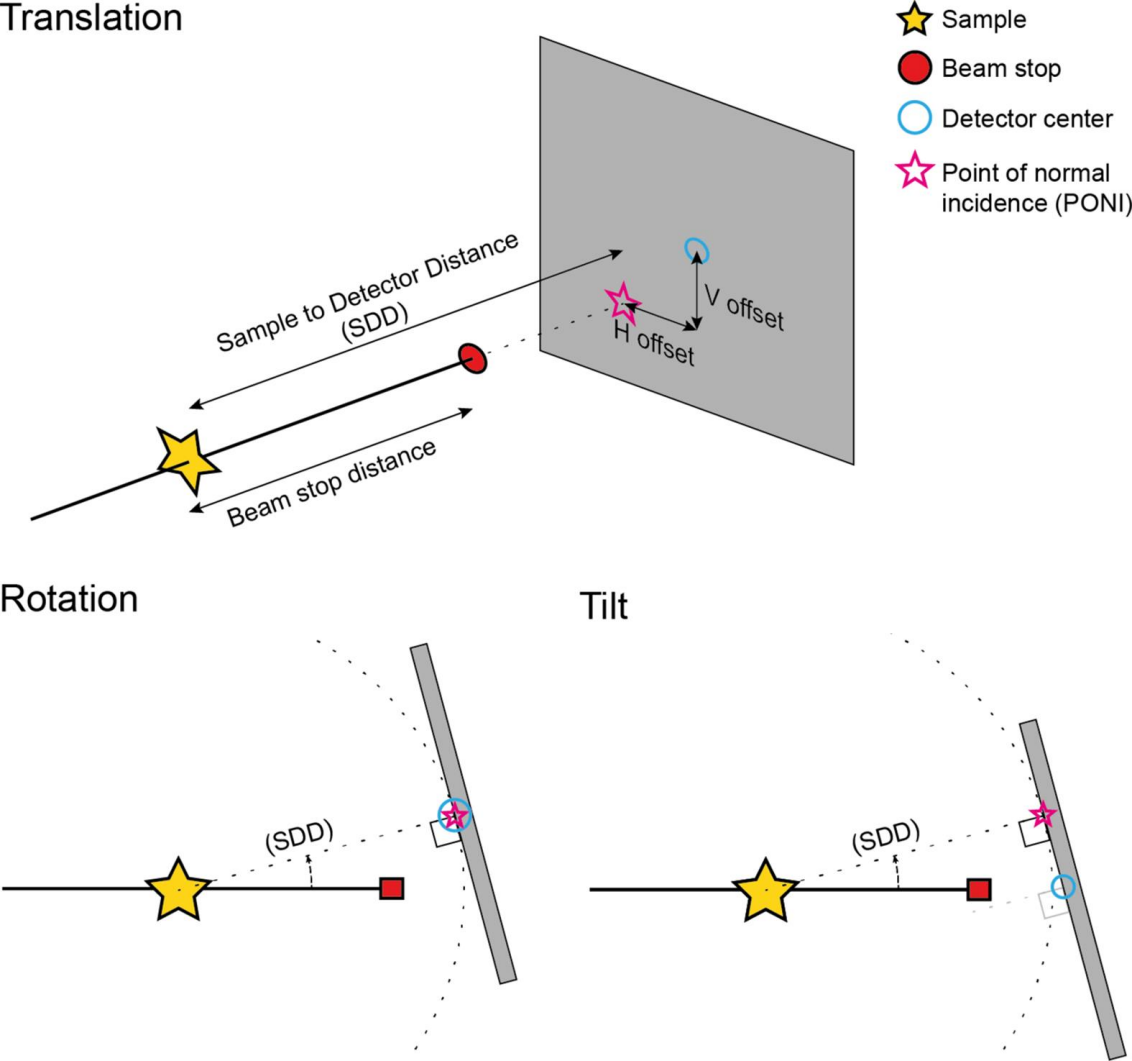
Geometry conventions used in *xrdPlanner*. Translations without any tilt/rotation are the horizontal and vertical distances between the centre of the detector and the PONI (top). The SDD is the distance from the sample to the PONI. A rotation moves the detector along the goniometer circle (constant SDD), keeping the PONI at the same position relative to the detector surface, here the detector centre (lower left). A tilt rolls the detector surface on the goniometer circle, hence the SDD is fixed, but the PONI shifts along the detector face (lower right).

The geometry (see Fig. 4 ▸) is defined with the centre of rotation at the sample position, such that the radius of the rotation circle is equal to the SDD. That is, the rotation moves the detector along the goniometer circle, keeping the point of normal incidence (PONI) at a constant position relative to the detector surface. At 0°, the detector surface is perpendicular to the beam and, at 90°, the detector surface is parallel with the beam with the detector normal pointing down. The tilt angle is defined relative to the detector face and such that the PONI shifts along the detector face, keeping the SDD fixed. The detector face is thus always tangential to the goniometer circle. The tilt can intuitively be described as a rolling motion along the goniometer circle and is considered a convenience function as it is equivalent to the combination of rotation and *V*_offset_. Consequently, the vertical shift of the PONI position on the detector face is equal to the arclength of a section on the goniometer circle with an angular span equal to the tilt angle.

The beamstop is modelled as the projection of a circular disc onto the detector plane as seen from the sample position. The diameter of the circular disc and its distance to the sample position are specified, and it is always positioned with its rotation axis coaxially with the beam axis.

## 5. Conclusions

We have presented a versatile tool for planning PXRD and TS experiments with area detectors at synchrotron beamlines. The fast and intuitive interactions help users quickly decide on an experimental setup and make *xrdPlanner* a useful tool for planning their experiment layout.

The software is designed to be easy to use and focus is put on modelling the parts essential for the beam-time planning without any coding experience required. The design philosophy has been to simplify where possible, to speed up calculations, and to have software that can quickly process and visualize changes in the geometry, making live exploration feasible. Other tools capable of more sophisticated simulations are available, *e.g.* *pyFAI* (Kieffer *et al.*, 2020 ▸), *Dans_Diffraction* (Porter & Prestipino, 2023 ▸), *xrd_simulator* (Henningsson & Hall, 2023 ▸) and *XMAS* (Tamura, 2014 ▸); however, some coding is required to take full advantage of these tools.

The Python-based development offers the ability for users to contribute, allowing the program to grow and shape toward the needs of the community. The possibility to import pre-defined settings from different beamlines makes it a useful tool for the general user.

Moreover, it can provide guidance and training for students to visualize how the accessible part of reciprocal space depends on the detector model (size), the photon energy and the position of the detector.

## 6. Resources

The software is written for *Python3* and is tested with version 3.11. It is available at the Python Package Index (PyPI) repository and can be installed using the Python package installer (*pip*). *xrdPlanner* is 100% free and open source under the GNU General Public Licence V3.0. It depends on the *Numpy* (Harris *et al.*, 2020 ▸), *PyQt6*, *PyQtGraph* (Campagnola, 2023 ▸), *pyFAI* (Kieffer *et al.*, 2020 ▸) and *Dans_Diffraction* (Porter & Prestipino, 2023 ▸) Python packages. A project page for *xrdPlanner* exists at https://github.com/LennardKrause/xrdPlanner.
